# Impact of 25-Hydroxyvitamin D on the Prognosis of Acute Ischemic Stroke: Machine Learning Approach

**DOI:** 10.3389/fneur.2020.00037

**Published:** 2020-01-31

**Authors:** Chulho Kim, Sang-Hwa Lee, Jae-Sung Lim, Yerim Kim, Min Uk Jang, Mi Sun Oh, San Jung, Ju-Hun Lee, Kyung-Ho Yu, Byung-Chul Lee

**Affiliations:** ^1^Department of Neurology, Chuncheon Sacred Heart Hospital, Chuncheon, South Korea; ^2^Chuncheon Translational Research Center, College of Medicine, Hallym University, Chuncheon, South Korea; ^3^Department of Neurology, Hallym University Sacred Heart Hospital, Anyang, South Korea; ^4^Department of Neurology, Kangdong Sacred Heart Hospital, Seoul, South Korea; ^5^Department of Neurology, Dongtan Sacred Heart Hospital, Dongtan, South Korea; ^6^Department of Neurology, Kangnam Sacred Heart Hospital, Seoul, South Korea

**Keywords:** vitamin D, cerebral infarction, stroke, outcome assessment, machine learning

## Abstract

**Background and Purpose:** Vitamin D is a predictor of poor outcome for cardiovascular disease. We evaluated whether serum 25-hydroxyvitamin D level was associated with poor outcome in patients with acute ischemic stroke (AIS) using machine learning approach.

**Materials and Methods:** We studied a total of 328 patients within 7 days of AIS onset. Serum 25-hydroxyvitamin D level was obtained within 24 h of hospital admission. Poor outcome was defined as modified Rankin Scale score of 3–6. Logistic regression and extreme gradient boosting algorithm were used to assess association of 25-hydroxyvitamin D with poor outcome. Prediction performances were compared with area under ROC curve and F1 score.

**Results:** Mean age of patients was 67.6 ± 13.3 years. Of 328 patients, 59.1% were men. Median 25-hydroxyvitamin D level was 10.4 (interquartile range, 7.1–14.8) ng/mL and 47.2% of patients were 25-hydroxyvitamin D-deficient (<10 ng/mL). Serum 25-hydroxyvitamin D deficiency was a predictor for poor outcome in multivariable logistic regression analysis (odds ratio, 3.38; 95% confidence interval, 1.24–9.18, *p* = 0.017). Stroke severity, age, and 25-hydroxyvitamin D level were also significant predictors in extreme gradient boosting classification algorithm. Performance of extreme gradient boosting algorithm was comparable to those of logistic regression (AUROC, 0.805 vs. 0.746, *p* = 0.11).

**Conclusions:** 25-hydroxyvitamin D deficiency was highly prevalent in Korea and low 25-hydroxyvitamin D level was associated with poor outcome in patients with AIS. The machine learning approach of extreme gradient boosting was also useful to assess stroke prognosis along with logistic regression analysis.

## Introduction

Vitamin D is a prohormone synthesized by sun-exposure and dietary intake (1). Besides its role in bone integrity and calcium homeostasis (2), vitamin D status is also associated with cardiovascular morbidity and mortality (3–6). Vitamin D has a protective effect on endothelial function and vascular remodeling in experimental models (7, 8). One meta-analysis has shown that low vitamin D level is associated with 2.5-fold increase of the risk of ischemic stroke (9). However, the benefit of vitamin D supplementation in improving cardiovascular outcome remains controversial (10–12).

Several studies have suggested that low vitamin D is also associated with poor outcome in patients with acute ischemic stroke (AIS) (13–17). In these studies, logistic regression analysis alone was used to assess the relationship between low vitamin D status and prognosis of AIS patients. Logistic regression analysis should consider model complexity including interaction and model appropriateness using goodness of fit (18). Vitamin D is associated with several cardiovascular risk factors, including hypertension (19), insulin resistance (20), cerebral small vessel disease burden (21), stroke severity (16, 17), infarct volume (22), mood (23), and cognition (24) in previous studies. However, studies considering these interactions in logistic regression models are scarce.

Recently, machine learning (ML) has been found to be capable of accurately predicting prognosis in several disease categories, including cancer (25), cardiovascular disease (26), and psychiatric disorders (27). ML has advantage of being able to deal with large-size data and having several optimization technique, thus reducing the overfitting of training algorithms (28). In addition, several ML models such as random forest and extreme gradient boosting are tree-based feature selection ML algorithms that enable us to find non-linear relationship and interactions between independent variables more efficiently compared to logistic regression model (29). In this study on the association between vitamin D and stroke prognosis, we hypothesize that low vitamin D is associated with poor outcome in patients with AIS and that the predicting performance of extreme gradient boosting is superior to that of binary logistic regression for considering vitamin D with several risk factors.

## Materials and Methods

### Study Population

The present study was a single-center retrospective study that screened all patients with AIS within 7 days of symptom onset using prospectively collected hospital registry. From August 2013 to October 2015, a total of 738 AIS patients with a positive diffusion-weighted lesion on brain MRI were selected for screening. We excluded patients with prior history of stroke (*n* = 243) because dietary intake or physical activity known to be associated with vitamin D intake and biosynthesis could be affected by premorbid neurological status. Additionally, patients without 3-month outcome capture (*n* = 85) and laboratory measures including admission serum 25-hydroxyvitamin D level (*n* = 82) were excluded.

### Measurement of Serum 25-Hydroxyvitamin D Level

Vitamin D status was determined by serum 25-hydroxyvitamin D concentration, a major circulating form with a long circulating half-life (~3 weeks) (30). 25-hydroxyvitamin D level was measured within 24 h of admission using a radioimmunoassay kit (DiaSorin Liaison, Stillwater, MN, USA) with <10% of inter-assay coefficient of variation. The assay was standardized against NIST Standard Reference Material 2972 (NIST, Gaithersburg, ME, USA) and certified by the Centers for Disease Control and Prevention Vitamin D Standardization Program. 25-hydroxyvitamin D deficiency was defined when its concentration was <10 ng/mL following the criteria of Korean population study (31).

### Covariates

Definitions for vascular risk factors were based on our previous reports (32). Hypertension was defined if participants were taking antihypertensive medications or if their average sitting systolic blood pressure was 140/90 mmHg or more. Diabetes was defined if they were taking medical treatments for diabetes, if they had a fasting serum glucose level of 126 mg/dL or more, or if they had a non-fasting random serum glucose level of 200 mg/dL or more with corresponding symptoms of diabetes. Subjects were considered to have hyperlipidemia if they had a fasting total cholesterol level of 240 mg/dL or more or if they were being treated with a lipid-lowering agent. A current smoker was defined as a person smoking one or more cigarettes per day within the last 6 months. Stroke subtypes were classified as large artery atherosclerosis (LAA), small vessel occlusion (SVO), cardioembolism (CE), undetermined etiology (SUE), and other determined etiology (SOE) according to the Trial of Org 10172 in Acute Stroke Treatment (TOAST) criteria (33). Laboratory parameters included serum level of fasting blood sugar, total cholesterol, triglyceride, high-density lipoprotein, low-density lipoprotein, blood urea nitrogen, creatinine, and whole blood level of white blood cell count, hemoglobin, and platelet count.

### Outcomes

Primary outcome measure was 3-months poor functional outcome defined as a modified Rankin Scale (mRS) score of 3–6. Certified neurologists and nurses assessed the mRS score. Secondary outcome was F1 score of the prediction model (binary logistic regression vs. extreme gradient boosting) for AIS prognosis classification. F1 score is a measure of the model's accuracy. It represents harmonized mean of precision (positive predictive value) and recall (sensitivity) of the ML classifier.

### Statistical Analysis

Baseline characteristics according to tertiles of 25-hydroxyvitamin D were compared with analysis of variance or Kruskal Wallis test for continuous variables. For categorical variables, Pearson's χ^2^-test or Fisher's exact test was used. We performed univariate and multivariate logistic regression analyses for 3-months poor functional outcome (mRS 3–6) according to 25-hydroxyvitamin D status. All variables that were significant with *p* < 0.10 in univariable analysis, age, and sex were entered into multivariable models. Two-sided *p* < 0.05 was considered significant in multivariable analysis performed with R version 3.4.1 (the R Foundation for Statistical Computing).

### Machine Learning Algorithm

We used the Extreme Gradient Boosting (XGBoost) R package that could perform regression and classification task well. XGBoost is an ML algorithm widely used in various Kaggle contests because it can perform non-linear prediction well, deal with missing data, and prove that computational speed is faster than other ML methods (34). As previously mentioned, we labeled patient's outcome as good (mRS: 0–2) or poor (mRS: 3–6). All participants' dataset were randomly divided into 2 group: training (60% of 328 subjects) and testing (40%). Proportion of poor outcome in training and testing dataset was identically allocated using R cart package. Input features of this model were independent variables in the multivariate logistic regression model. Precise parameter tuning methods of XGBoost for preventing overfitting of this ML model are shown in Supplemental Material. We calculated the probabilistic score of the prediction model of binary logistic regression and extreme gradient boosting algorithm, in which we set the probabilistic score of 0.5 or more to “poor outcome” (or test positive) and those <0.5 to “good outcome.” We presented the performance of these two classification tasks as precision, recall, accuracy, and F1 score. We compared model performance with area under the ROC curve (AUROC). Additionally, we also examined the performance of other machine learning algorithms such as support vector machines.

## Results

### Baseline Characteristics According to 25-Hydroxyvitamin D Tertile

A total of 328 patients (mean age, 67.4 ± 13.2 years; male, 59.1%) were included in this study (Table 1). The mean (± standard deviation) and median [interquartile range (IQR)] of 25-hydroxyvitamin D were 11.5 ± 6.1 and 10.3 (7.1–14.7) ng/mL, respectively. The 25-hydroxyvitamin D tertile was inversely correlated with NIHSS score (*p* for Kruskal Wallis test = 0.029) and blood urea nitrogen level (*p* for one-way ANOVA = 0.007). It was negatively correlated with hemoglobin level (*p* for one-way ANOVA < 0.001). Stroke subtype and cardiovascular risk factors such as hypertension, diabetes, smoking, or atrial fibrillation showed no significant associations with 25-hydroxyvitamin D tertile.

**Table 1:** Baseline characteristic of participants according to 25-hydroxyvitamin D tertile.

	**Total**	**25-hydroxyvitamin D concentration**			***p***
		**Tertile 1 (*n* = 109, 3.0–7.8 ng/mL)**	**Tertile 2 (*n* = 109, 7.9–12.6 ng/mL)**	**Tertile 3 (*n* = 110, 12.7–37.4 ng/mL)**	
Age (±SD), years	67.4 ± 13.2	69.5 ± 14.0	66.2 ± 13.1	66.7 ± 12.4	0.138
Male, %	194 (59.1)	58 (53.2)	65 (59.6)	71 (64.5)	0.231
BMI, IQR, Kg/m^2^	24.0 ± 3.4	24.2 ± 4.4	23.8 ± 2.7	23.9 ± 2.8	0.720
Hypertension, %	220 (67.1)	79 (72.5)	75 (68.8)	66 (60.0)	0.130
Diabetes, %	104 (31.7)	42 (38.5)	32 (29.4)	30 (27.3)	0.164
Hyperlipidemia, %	116 (35.4)	42 (38.5)	41 (37.6)	33 (30.0)	0.349
Current smoking, %	138 (42.1)	41 (37.6)	45 (41.3)	52 (47.3)	0.344
Atrial fibrillation, %	65 (19.8)	20 (18.3)	23 (21.1)	22 (20.0)	0.877
TOAST classification					0.469
LAA	124 (37.8)	35 (32.1)	46 (42.2)	43 (39.1)	
SVO	94 (28.7)	31 (28.4)	29 (26.6)	34 (30.9)	
CE	65 (19.8)	22 (20.2)	22 (20.2)	21 (19.1)	
SOE and SUE	45 (13.7)	21 (19.3)	12 (11.0)	12 (10.9)	
NIHSS score, mean ± SD	5.4 ± 6.0	6.6 ± 7.1	5.5 ± 5.6	4.2 ± 5.0	0.012
Median(IQR)	3.0 (1.0–8.8)	3.0 (1.5–9.5)	3.0 (1.5–11.0)	2.0 (1.0–5.3)	0.029
IV thrombolysis	44 (13.4)	13 (11.9)	20 (18.3)	11 (10.0)	0.166
Fasting blood glucose, mg/dL	125 ± 53	131 ± 62	118 ± 39	124 ± 54	0.217
Total cholesterol, mg/dL	181 ± 40	177 ± 47	182 ± 42	185 ± 31	0.281
TG, mg/dL	121 ± 74	126 ± 82	110 ± 58	125 ± 78	0.205
HDL, mg/dL	49 ± 16	47 ± 15	49 ± 16	51 ± 17	0.102
LDL, mg/dL	115 ± 37	111 ± 43	116 ± 38	117 ± 29	0.377
BUN, mg/dL	16.9 ± 8.7	19.1 ± 10.8	15.8 ± 8.5	15.9 ± 5.7	0.007
Creatinine, mg/dL	0.9 ± 1.1	1.1 ± 1.3	0.8 ± 0.4	0.9 ± 1.3	0.206
eGFR, mL/min/1.73 m^2^	62.3 ± 84.9	69.2 ± 97.0	52.5 ± 23.2	65.3 ± 107.8	0.313
WBC count, × 10^3^ cells/mm^3^	8.2 ± 3.3	8.3 ± 3.0	8.0 ± 2.4	8.3 ± 4.2	0.803
Hemoglobin, g/dL	13.6 ± 2.1	13.0 ± 2.4	13.8 ± 1.9	14.1 ± 1.7	<0.001
Platelet count, × 10^3^/L	227 ± 77	231 ± 85	235 ± 90	215 ± 50	0.146
BPsystolic, mmHg	148 ± 26	149 ± 26	147 ± 27	148 ± 26	0.751
BPdiastolic, mmHg	85 ± 16	85 ± 15	85 ± 16	85 ± 16	0.944

*BMI, body mass index; IQR, interquartile range; TOAST, Trial of Org 10172 in Acute Stroke Treatment; LAA, large artery atherosclerosis; SVO, small vessel occlusion; CE, cardioembolism; SOE, stroke of other determined etiology; SUE, stroke of undetermined etiology; NIHSS, National Institute of Health Stroke Scale; IV, intravenous; TG, triglyceride; HDL, high-density lipoprotein; LDL, low-density lipoprotein; BUN, blood urea nitrogen; eGFR, estimated glomerular filtration rate; WBC, white blood cell; BP, blood pressure*.

### 25-Hydroxyvitamin D Level and Poor Outcome

**Figure 1:**
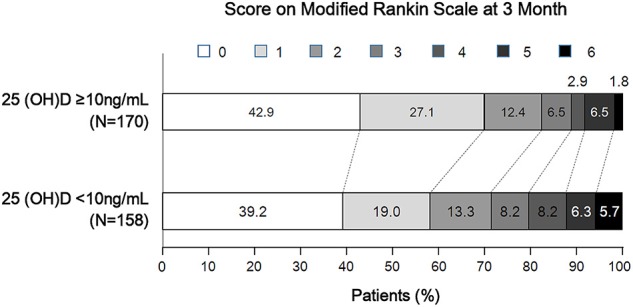
Distribution of 3-month modified Rankin Scale score according to 25-hydroxyvitamin D deficiency status.

Of 328 participants, proportions of AIS patients with poor 3-months outcome and 25-hydroxyvitamin D deficiency were 22.9 and 48.2%, respectively (Table 2). Patients with 25-hydroxyvitamin D deficiency tended to have higher mRS score compared to those without such deficiency (p for χ^2^ trend = 0.026, Figure 1). Poor outcome at 3-months was positively associated with age, female gender, stroke severity, blood urea nitrogen, and white blood cell count. Hemoglobin level and 25-hydroxyvitamin D deficiency were inversely associated with poor outcome in patients with AIS. In univariate analysis, 25-hydroxyvitamin D deficiency was a predictor for poor 3-months outcome [OR (odds ratio), 1.86; CI (confidence interval), 1.10–3.14; *p* = 0.021]. Although age, sex, stroke severity, stroke subtype, intravenous thrombolysis, blood urea nitrogen, white blood cell count, and hemoglobin were adjusted in the multivariate logistic regression model, 25-hydroxyvitamin D deficiency was a significant predictor for 3-months poor outcome (OR, 3.21; CI, 1.22–8.48; *p* = 0.019, Table 3).

**Table 2:** Differences of demographic and clinical characteristics between good and poor outcome groups of patients with acute ischemic stroke.

	**Poor (*n* = 75)**	**Good (*n* = 253)**	**Total (*n* = 328)**	***p***
Age, years	72.6 ± 14.3	65.9 ± 12.5	67.4 ± 13.2	<0.001
Male, %	37 (49.3)	157 (62.1)	194 (59.1)	0.049
BMI, Kg/m^2^	24.2 ± 5.1	23.9 ± 2.7	24.0 ± 3.4	0.628
Hypertension, %	54 (72.0)	166 (65.6)	220 (67.1)	0.301
Diabetes, %	25 (33.3)	79 (31.2)	104 (31.7)	0.730
Hyperlipidemia, %	29 (38.7)	87 (34.4)	116 (35.4)	0.496
Current smoking, %	27 (36.0)	111 (43.9)	138 (42.1)	0.225
Atrial fibrillation, %	19 (25.3)	46 (18.2)	65 (19.8)	0.172
TOAST classification				0.329
LAA	27 (36.0)	97 (38.3)	124 (37.8)	
SVO	17 (22.7)	77 (30.4)	94 (28.7)	
CE	17 (22.7)	48 (19.0)	65 (19.8)	
SOE and SUE	14 (18.7)	31 (12.3)	45 (13.7)	
NIHSS, mean ± SD	10.2 ± 7.0	4.0 ± 4.9	5.4 ± 6.0	<0.001
NIHSS, median (IQR)	9 (4-15)	2 (1-5)	3.0 (1.0–8.8)	<0.001
IV thrombolysis	15 (20.0)	29 (11.5)	44 (13.4)	0.057
Fasting blood glucose, mg/dL	131 ± 57	123 ± 52	125 ± 53	0.240
Total cholesterol, mg/dL	187 ± 47	180 ± 38	181 ± 40	0.195
TG, mg/dL	110 ± 82	124 ± 71	121 ± 74	0.154
HDL, mg/dL	52 ± 14	48 ± 16	49 ± 16	0.120
LDL, mg/dL	119 ± 45	113 ± 35	115 ± 37	0.252
BUN, mg/dL	18.9 ± 8.7	16.3 ± 8.6	16.9 ± 8.7	0.026
Creatinine, mg/dL	0.9 ± 0.4	1.0 ± 1.2	0.9 ± 1.1	0.456
WBC count, × 10^3^ cells/mm^3^	8.9 ± 3.2	8.0 ± 3.3	8.2 ± 3.3	0.032
Hemoglobin, g/dL	13.2 ± 2.4	13.8 ± 2.0	13.6 ± 2.1	0.040
Platelet count, × 10^3^/L	234 ± 92	225 ± 72	227 ± 77	0.378
BPsystolic, mmHg	149 ± 26	147 ± 26	148 ± 26	0.613
BPdiastolic, mmHg	83 ± 15	86 ± 16	85 ± 16	0.288
25-hydroxyvitamin D deficiency, %	45 (60.0)	113 (44.7)	158 (48.2)	0.020

*BMI, body mass index; TOAST, Trial of Org 10172 in Acute Stroke Treatment; LAA, large artery atherosclerosis; SVO, small vessel occlusion; CE, cardioembolism; SOE, stroke of other determined etiology; SUE, stroke of undetermined etiology; NIHSS, National Institute of Health Stroke Scale; IQR, interquartile range; IV, intravenous; TG, triglyceride; HDL, high-density lipoprotein; LDL, low-density lipoprotein; BUN, blood urea nitrogen; eGFR, estimated glomerular filtration rate; WBC, white blood cell; BP, blood pressure*.

**Table 3:** Binary logistic regression analysis of predictors for poor functional outcome (mRS 3-6) at 3 months.

	**OR (95% CI)**	***p***
25(OH)D deficiency (<10 ng/mL)	1.86 (1.10–3.14)	0.021
Model 1	1.74 (1.01–2.97)	0.044
Model 2	3.01 (1.16–7.84)	0.024
Model 3	3.18 (1.21–8.36)	0.019
Model 4	3.21 (1.22–8.48)	0.019

mRS, modified Rankin Scale; OR, odds ratio; CI, confidence interval; 25(OH)D, 25-hydroxyvitamin D. Model 1 was adjusted for age and sex. Model 2 included variables in model 1 plus NIHSS and NIHSS^*^25-hydroxyvitamin D deficiency interaction term. Model 3 included variables in model 2 plus stroke subtype (TOAST classification) and intravenous thrombolysis. Model 4 included variables in model 3 plus blood urea nitrogen, white blood cell count, and hemoglobin.

### Comparison of Logistic Regression and the Other Machine Learning Classifier

**Figure 2:**
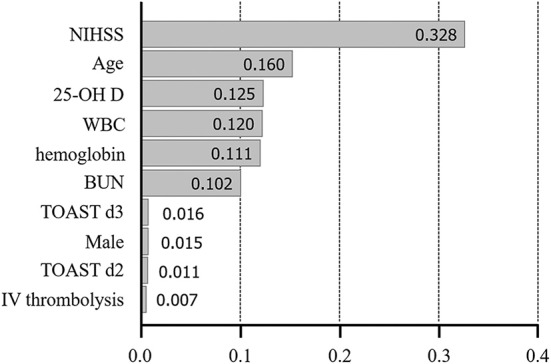
Variable importance plot for XGBoost. NIHSS, National Institute of Health Stroke Scale; 25-OH D, 25-hydroxyvitamin D, WBC, white blood cell; BUN, blood urea nitrogen; TOAST, Trial of Org 10172 in Acute Stroke Treatment; LAA, large artery atherosclerosis; d2-3, 2nd and 3rd dummy variable; IV, intravenous. X-axis represents variable importance in the extreme gradient boosting prediction.

**Figure 3:**
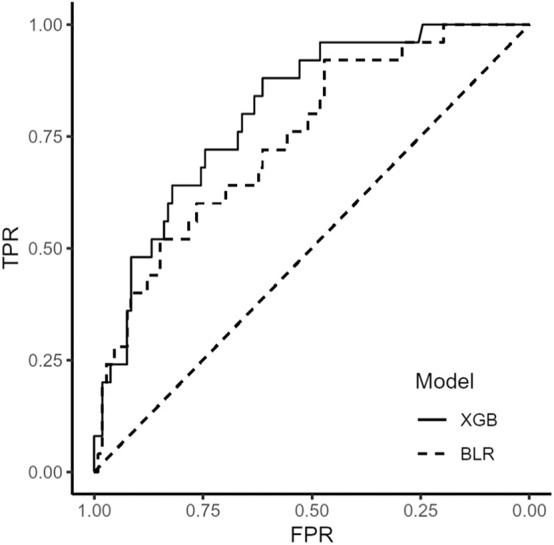
Receiver operating characteristic curve of binary logistic regression and extreme gradient boosting algorithm. BLR, binary logistic regression; XGB, extreme gradient boosting.

We used extreme gradient boosting ML algorithm to classify and predict factors associated with poor 3-months outcome of AIS. Figure 2 shows factors affecting this binary classification weighed by XGBoost algorithm. NIHSS (feature importance, 32.8%) score was the top predictive factor affecting XGBoost classification, followed by age (16.0%) and 25-hydroxyvitamin D level (12.5%) in the training dataset. Table 4 shows result of classification performance of binary logistic regression and extreme gradient boosting to predict 3-months poor outcome. Overall prediction performances including precision, recall, accuracy, and F1 score were higher in extreme gradient boosting model compared to those in the binary logistic regression model. Figure 3 shows performances of these two classifiers with AUROC curve analysis for the test dataset (total 131 subjects). The XGBoost's performance was higher than those of logistic regression in classifying prognosis of patients with AIS, but statistical significance was not achieved (AUROC, 0.805 vs. 0.746, *p*-value = 0.11). We also examined the performance of the support vector machine for the binary prediction of poor outcome, and overall procedures of the support vector machine was presented in the Supplemental Material.

**Table 4:** Results of classification performance of binary logistic regression and extreme gradient boosting to predict 3-month poor outcome of AIS patients in the test data.

	**TP**	**FP**	**FN**	**TN**	**Total**	**Recall**	**Specificity**	**Precision**	**NPV**	**Accuracy**	**F1 score**
BLR	10	11	15	95	131	40.0	89.6	47.6	86.4	80.2	43.5
XGB	12	10	13	96	131	48.0	90.6	54.5	88.1	82.4	51.1

*TP, true positive; FP, false positive; FN, false negative; TN, true negative; NPV, negative predictive value; BLR, binary logistic regression; XGB, extreme gradient boosting*.

## Discussion

Our study ascertained that low 25-hydroxyvitamin D status in stroke patients was highly prevalent even at the time of admission of AIS patients and that 25-hydroxyvitamin D status was a significant predictor for poor outcome after adjusting multiple variables in logistic regression model. Additionally, serum 25-hydroxyvitamin D level was also associated with a poor outcome in the XGBoost ML model that considered multiple interactions.

Are there possible causal relationship between vitamin D deficiency and stroke prognosis? First, it has been shown that endothelial dysfunction has an important role in atherosclerosis and stroke development (35, 36). Markers for endothelial dysfunction are associated with stroke lesion volume or clinical outcome (37). Witham et al. have reported that high dose vitamin D supplementation for 16 weeks can increase flow mediated dilatation in patients with stroke (38) which is one of well-known predictors for the cardiovascular outcome (39). These could explain a possible link between vitamin D status and stroke outcome. Second, low vitamin D status is closely related to “general health status” which could reflect individual's physical activity or dietary intake. On the contrary, vitamin D status can predict physical performance in an aged populations (40). Krarup et al. have reported that pre-stroke physical activity is associated with severity and long-term outcome from first-ever ischemic stroke (41). In this regards, low 25-hydroxyvitamin D could be a result of poor general health or increased frailty in these stroke population. New interventional studies on 25-hydroxyvitamin D supplementation in AIS patients with low vitamin D status are needed to improve our understanding of role of 25-hydroxyvitamin D in cardiovascular disease.

We observed that 25-hydroxyvitamin D levels were extremely low in our stroke population compared to the normal Korean population (31). If so, how can we explain these high prevalence of 25-hydroxyvitamin D deficiency or insufficiency in stroke participants? First, the prevalence of 25-hydroxyvitamin D deficiency has been gradually increasing as more people are living in a modernized and indoor place in which sun light exposure is insufficient to produce vitamin D biosynthesis. Analysis of national health surveys in US could support this hypothesis (42). Second, relatively high prevalence of vitamin D deficiency in stroke patients has been reported in other cohorts (43, 44). In these studies, mean level of 25-hydroxyvitamin D concentration ranged from 13.7 to 14.2 ng/mL, comparable to that of our study. Unquestionably, prevalence of vitamin D deficiency is a pandemic phenomenon. It has markedly increased irrespective of age, sex, ethnicity, types of concurrent comorbidity, or socioeconomic state.

Despite of these positive correlation between 25-hydroxyvitamin D deficiency and various cardiovascular outcomes, there have been few evidence of randomized controlled trials whether vitamin D supplementation has a positive effect on the cardiovascular outcome (45). These discrepancies could be partly explained by various factors such as supplemental dosage of vitamin D, ethnical and environmental factors for the study participants, study design, and outcome in each trials. It's too early to conclude that vitamin D deficiency is as salient predictor for cardiovascular disease. Therefore, future trial are necessary to consider these differences and the exact causal pathways of vitamin D metabolism. We used logistic regression and XGBoost ML algorithm to predict stroke outcome in this study. Logistic regression is the most popular multivariate statistical method in medical science. Using logistic regression, we can quantitatively estimate the relationship between two variables of interest through an index called “odds ratio.” However, it is impossible to consider all interactions among variables in logistic regression because there might be a variety of combinations of interactions between variables in the real world. Results of XGBoost ML algorithm showed that, in addition to important predictors such as stroke and stroke severity, factors such as 25-hydroxyvitamin D, white blood cell count, and hemoglobin were also important factors predicting prognosis of stroke patients. We could suggest that 25-hydroxyvitamin D is associated with a variety of factors affecting stroke prognosis such as stroke severity, age, several laboratory parameters, stroke subtype, and gender and that XGBoost ML algorithm is useful for dealing with multiple interactions between independent predictors in binary classification of stroke prognosis. In addition, XGBoost algorithm can analyze large size data with a high computational speed. In this era, medical data are becoming so vast that it becomes hard to analyze. An ML approach can be an expeditious and efficient way to analyze such big data.

Our study has several limitations. First, our study was a single center retrospective case control study, although measurement of 25-hydroxyvitamin D level and 3-months clinical outcome capture were prospectively collected. Therefore, our results could not presume a causal relationship of vitamin D and stroke severity. In addition, they could not be generalizable to entire ischemic stroke patients or other ethnicities. Second, although pre-stroke physical activity and dietary intake were significant confounders in assessment with vitamin D level and stoke severity, we could not assess these variables in our model. However, some strengths of our study could reinforce the relationship between vitamin D status and stroke outcome. First, we excluded patients with prior history of stroke to minimize the effect of physical activity and dietary intake. Second, analysis of vitamin D and stroke outcome was adjusted for clinically important variables with a multivariate logistic model. Lastly, interactions between all independent variables were statistically considered by means of an ensemble ML mechanism called extreme gradient boosting.

In conclusion, 25-hydroxyvitamin D level is associated with 3-months poor outcome in patients with AIS. In addition, extreme gradient boosting ML algorithm could reveal the association of stroke prognosis with 25-hydroxyvitamin D level which might have certain interactions with other predictors.

## Data Availability Statement

The datasets analyzed in this article are not publicly available. Requests to access the datasets should be directed to the corresponding author (gumdol52@hallym.or.kr).

## Ethics Statement

The studies involving human participants were reviewed and approved by Hallym University Sacred Heart Hospital Institutional Review Board/Ethics Committee (IRB No. 2015-I064). The patients/participants provided their written informed consent to participate in this study.

## Author Contributions

CK and B-CL drafted the manuscript. J-HL performed the statistical analysis. S-HL and MO contributed to the statistical analyses. J-SL and YK provided critical suggestions for the discussion. MJ and SJ revised the first draft. K-HY designed the analyses to the final manuscript and technical comments on the results.

### Conflict of Interest

The authors declare that the research was conducted in the absence of any commercial or financial relationships that could be construed as a potential conflict of interest.

## References

[1] Rajakumar K. Vitamin D, cod-liver oil, sunlight, and rickets: a historical perspective. Pediatrics. (2003) 112:e132–5. 10.1542/peds.112.2.e132 12897318

[2] Khazai N Judd SE Tangpricha V. Calcium and vitamin D: skeletal and extraskeletal health. Curr Rheumatol Rep. (2008) 10:110–7. 10.1007/s11926-008-0020-y 18460265 PMC2669834

[3] Giovannucci E Liu Y Hollis BW Rimm EB. 25-hydroxyvitamin D and risk of myocardial infarction in men: a prospective study. Arch Intern Med. (2008) 168:1174–80. 10.1001/archinte.168.11.1174 18541825 PMC3719391

[4] Deleskog A Piksasova O Silveira A Samnegard A Tornvall P Eriksson P. Serum 25-hydroxyvitamin D concentration, established and emerging cardiovascular risk factors and risk of myocardial infarction before the age of 60 years. Atherosclerosis. (2012) 223:223–9. 10.1016/j.atherosclerosis.2012.04.014 22652526

[5] Correia LC Sodre F Garcia G Sabino M Brito M Kalil F. Relation of severe deficiency of vitamin D to cardiovascular mortality during acute coronary syndromes. Am J Cardiol. (2013) 111:324–7. 10.1016/j.amjcard.2012.10.006 23174181

[6] Skaaby T Husemoen LL Pisinger C Jorgensen T Thuesen BH Fenger M. Vitamin D status and incident cardiovascular disease and all-cause mortality: a general population study. Endocrine. (2013) 43:618–25. 10.1007/s12020-012-9805-x 23015273

[7] Hamabe A Takase B Uehata A Kurita A Ohsuzu F Tamai S. Impaired endothelium-dependent vasodilation in the brachial artery in variant angina pectoris and the effect of intravenous administration of vitamin C. Am J Cardiol. (2001) 87:1154–9. 10.1016/S0002-9149(01)01485-0 11356389

[8] Al Mheid I Patel R Murrow J Morris A Rahman A Fike L. Vitamin D status is associated with arterial stiffness and vascular dysfunction in healthy humans. J Am Coll Cardiol. (2011) 58:186–92. 10.1016/j.jacc.2011.02.051 21718915 PMC3896949

[9] Zhou R Wang M Huang H Li W Hu Y Wu T. Lower vitamin D status is associated with an increased risk of ischemic stroke: a systematic review and meta-analysis. Nutrients. (2018) 10:E277. 10.3390/nu10030277 29495586 PMC5872695

[10] Ford JA MacLennan GS Avenell A Bolland M Grey A Witham M. Cardiovascular disease and vitamin D supplementation: trial analysis, systematic review, and meta-analysis. Am J Clin Nutr. (2014) 100:746–55. 10.3945/ajcn.113.082602 25057156

[11] Wang L Song Y Manson JE Pilz S Marz W Michaelsson K. Circulating 25-hydroxy-vitamin D and risk of cardiovascular disease: a meta-analysis of prospective studies. Circ Cardiovasc Qual Outcomes. (2012) 5:819–29. 10.1161/CIRCOUTCOMES.112.967604 23149428 PMC3510675

[12] Elamin MB Abu Elnour NO Elamin KB Fatourechi MM Alkatib AA Almandoz JP. Vitamin D and cardiovascular outcomes: a systematic review and meta-analysis. J Clin Endocrinol Metab. (2011) 96:1931–42. 10.1210/jc.2011-0398 21677037

[13] Park KY Chung PW Kim YB Moon HS Suh BC Won YS. Serum vitamin D status as a predictor of prognosis in patients with acute ischemic stroke. Cerebrovasc Dis. (2015) 40:73–80. 10.1159/000434691 26184826

[14] Yalbuzdag SA Sarifakioglu B Afsar SI Celik C Can A Yegin T. Is 25(OH)D associated with cognitive impairment and functional improvement in stroke? A retrospective clinical study. J Stroke Cerebrovasc Dis. (2015) 24:1479–86. 10.1016/j.jstrokecerebrovasdis.2015.03.007 25922112

[15] Daubail B Jacquin A Guilland JC Khoumri C Aboa-Eboule C Giroud M. Association between serum concentration of vitamin D and 1-year mortality in stroke patients. Cerebrovasc Dis. (2014) 37:364–7. 10.1159/000362534 24970287

[16] Wang Y Ji H Tong Y Zhang ZB. Prognostic value of serum 25-hydroxyvitamin D in patients with stroke. Neurochem Res. (2014) 39:1332–7. 10.1007/s11064-014-1316-0 24789365

[17] Daubail B Jacquin A Guilland JC Hervieu M Osseby GV Rouaud O. Serum 25-hydroxyvitamin D predicts severity and prognosis in stroke patients. Eur J Neurol. (2013) 20:57–61. 10.1111/j.1468-1331.2012.03758.x 22632854

[18] Hosmer DW Taber S Lemeshow S. The importance of assessing the fit of logistic regression models: a case study. Am J Public Health. (1991) 81:1630–5. 10.2105/AJPH.81.12.1630 1746660 PMC1405276

[19] Afzal S Nordestgaard BG. Vitamin D, hypertension, and ischemic stroke in 116 655 individuals from the general population: a genetic study. Hypertension. (2017). 10.1161/HYPERTENSIONAHA.117.09411. [Epub ahead of print]. 28760944

[20] Pittas AG Harris SS Stark PC Dawson-Hughes B. The effects of calcium and vitamin D supplementation on blood glucose and markers of inflammation in nondiabetic adults. Diabetes Care. (2007) 30:980–6. 10.2337/dc06-1994 17277040

[21] Feng C Tang N Huang H Zhang G Qi X Shi F. 25-Hydroxy vitamin D level is associated with total MRI burden of cerebral small vessel disease in ischemic stroke patients. Int J Neurosci. (2019) 129:49–54. 10.1080/00207454.2018.1503182 30033803

[22] Turetsky A Goddeau RP Henninger N. Low serum vitamin D is independently associated with larger lesion volumes after ischemic stroke. J Stroke Cerebrovasc Dis. (2015) 24:1555–63. 10.1016/j.jstrokecerebrovasdis.2015.03.051 26009498

[23] Parker GB Brotchie H Graham RK. Vitamin D and depression. J Affect Disord. (2017) 208:56–61. 10.1016/j.jad.2016.08.082 27750060

[24] Sari A Durmus B Karaman CA Ogut E Aktas I. A randomized, double-blind study to assess if vitamin D treatment affects the outcomes of rehabilitation and balance in hemiplegic patients. J Phys Ther Sci. (2018) 30:874–8. 10.1589/jpts.30.874 29950783 PMC6016314

[25] Sepehri S Upadhaya T Desseroit M-C Visvikis D Le Rest CC Hatt M Comparison of machine learning algorithms for building prognostic models in non-small cell lung cancer using clinical and radiomics features from 18F-FDG PET/CT images. J Nucl Med. (2018) 59:328.

[26] Weng SF Reps J Kai J Garibaldi JM Qureshi N. Can machine-learning improve cardiovascular risk prediction using routine clinical data? PLoS ONE. (2017) 12:e0174944. 10.1371/journal.pone.0174944 28376093 PMC5380334

[27] Janssen RJ Mourão-Miranda J Schnack HG. Making individual prognoses in psychiatry using neuroimaging and machine learning. Biol Psychiatry Cogn Neurosci Neuroimaging. (2018) 3:798–808. 10.1016/j.bpsc.2018.04.004 29789268

[28] Gupta O Das AJ Hellerstein J Raskar R. Machine learning approaches for large scale classification of produce. Sci Rep. (2018) 8:5226. 10.1038/s41598-018-23394-3 29588477 PMC5869718

[29] Zhou S AbdelWahab A Sapp JL Warren JW Horáček BM. Localization of ventricular activation origin from the 12-lead ECG: a comparison of linear regression with non-linear methods of machine learning. Ann Biomed Eng. (2019) 47:403–12. 10.1007/s10439-018-02168-y 30465152

[30] Lips P. Which circulating level of 25-hydroxyvitamin D is appropriate? J Steroid Biochem Mol Biol. (2004) 89:611–4. 10.1016/j.jsbmb.2004.03.040 15225848

[31] Choi HS Oh HJ Choi H Choi WH Kim JG Kim KM. Vitamin D insufficiency in Korea—a greater threat to younger generation: the Korea National Health and Nutrition Examination Survey (KNHANES) 2008. J Clin Endocrinol Metab. (2011) 96:643–51. 10.1210/jc.2010-2133 21190984

[32] Kim C Jang MU Oh MS Park JH Jung S Lee JH. Off-hour effect on 3-month functional outcome after acute ischemic stroke: a prospective multicenter registry. PLoS ONE. (2014) 9:e105799. 10.1371/journal.pone.0105799 25165816 PMC4148337

[33] Adams HP Bendixen BH Kappelle LJ Biller J Love BB Gordon DL. Classification of subtype of acute ischemic stroke. Definitions for use in a multicenter clinical trial. TOAST. Trial of Org 10172 in Acute Stroke Treatment. Stroke. (1993) 24:35–41. 10.1161/01.STR.24.1.35 7678184

[34] Mangal A Kumar N editors. Using big data to enhance the bosch production line performance: a Kaggle challenge. In: 2016 IEEE International Conference on Big Data. Washington, DC (2016). p. 2029–35. 10.1109/BigData.2016.7840826

[35] Choy JC Granville DJ Hunt DW McManus BM. Endothelial cell apoptosis: biochemical characteristics and potential implications for atherosclerosis. J Mol Cell Cardiol. (2001) 33:1673–90. 10.1006/jmcc.2001.1419 11549346

[36] Jung KH Chu K Lee ST Park HK Bahn JJ Kim DH. Circulating endothelial microparticles as a marker of cerebrovascular disease. Ann Neurol. (2009) 66:191–9. 10.1002/ana.21681 19743467

[37] Simak J Gelderman MP Yu H Wright V Baird AE. Circulating endothelial microparticles in acute ischemic stroke: a link to severity, lesion volume and outcome. J Thromb Haemost. (2006) 4:1296–302. 10.1111/j.1538-7836.2006.01911.x 16706974

[38] Witham M Dove F Sugden J Doney A Struthers A. The effect of vitamin D replacement on markers of vascular health in stroke patients–A randomised controlled trial. Nutr Metab Cardiovasc Dis. (2010) 22:864–70. 10.1016/j.numecd.2010.11.001 21194910

[39] Inaba Y Chen JA Bergmann SR. Prediction of future cardiovascular outcomes by flow-mediated vasodilatation of brachial artery: a meta-analysis. Int J Cardiovasc Imaging. (2010) 26:631–40. 10.1007/s10554-010-9616-1 20339920

[40] Wicherts IS van Schoor NM Boeke AJP Visser M Deeg DJ Smit J. Vitamin D status predicts physical performance and its decline in older persons. J Clin Endocrinol Metab. (2007) 92:2058–65. 10.1210/jc.2006-1525 17341569

[41] Krarup L-H Truelsen T Gluud C Andersen G Zeng X Korv J. Prestroke physical activity is associated with severity and long-term outcome from first-ever stroke. Neurology. (2008) 71:1313–8. 10.1212/01.wnl.0000327667.48013.9f 18936423

[42] Ginde AA Liu MC Camargo CA. Demographic differences and trends of vitamin D insufficiency in the US population, 1988–2004. Arch Intern Med. (2009) 169:626–32. 10.1001/archinternmed.2008.604 19307527 PMC3447083

[43] Chung PW Park KY Kim JM Shin DW Park MS Chung YJ. 25-hydroxyvitamin D status is associated with chronic cerebral small vessel disease. Stroke. (2015) 46:248–51. 10.1161/STROKEAHA.114.007706 25424481

[44] Tu W-J Zhao S-J Xu D-J Chen H. Serum 25-hydroxyvitamin D predicts the short-term outcomes of Chinese patients with acute ischaemic stroke. Clin Sci. (2014) 126:339–46. 10.1042/CS20130284 24020395

[45] Saponaro F Marcocci C Zucchi R. Vitamin D status and cardiovascular outcome. J Endocrinol Invest. (2019) 42:1285–90. 10.1007/s40618-019-01057-y 31172459

