# Supplementary material for: Impact of 25-Hydroxyvitamin D on the Prognosis of Acute Ischemic Stroke: Machine Learning Approach
Source: Front Neurol. 2020 Jan 31;11:37. doi: 10.3389/fneur.2020.00037 (PMC7005206; doi:10.3389/fneur.2020.00037)
Supplement: Supplementary file 1 [file Table_1.DOCX]

| Parameter | Value |
| --- | --- |
| objective function | binary logistic |
| number of iteration (nrounds) | 20 |
| learning rate (eta) | 0.3 |
| evaluation | logloss |
| maximal depth | 5 |
| minimum loss reduction (gamma) | 0 |

**Supplemental Table 1.** Parameters using extreme gradient boosting model.
