# Supplementary material for: Impact of 25-Hydroxyvitamin D on the Prognosis of Acute Ischemic Stroke: Machine Learning Approach
Source: Front Neurol. 2020 Jan 31;11:37. doi: 10.3389/fneur.2020.00037 (PMC7005206; doi:10.3389/fneur.2020.00037)
Supplement: Supplementary file 2 [file Table_2.DOCX]

**Supplemental methods**

We also examined the performance of the support vector machine.^1^ In brief, there is the plane divides the two classes in the data is called the separation hyperplane. Support vector machine is an algorithm that selects the best separation hyperplane from the two support vector, which is the margin of the data for the classification.^2^ We used 10-fold cross-validation, linear kernel and optimized parameter tuning for the gamma and cost values. Result of the support vector machine showed in the following table, which was similar to that of binary logistic regression.

**Supplemental Table.** Results of classification performance of binary logistic regression and support vector machine to predict 3-month poor outcome of AIS patients in the test data.

|  | TP | FP | FN | TN | Total | Recall | Specificity | Precision | NPV | Accuracy | F1 score |
| --- | --- | --- | --- | --- | --- | --- | --- | --- | --- | --- | --- |
| BLR | 10 | 11 | 15 | 95 | 131 | 40.0 | 89.6 | 47.6 | 86.4 | 80.2 | 43.5 |
| SVM | 13 | 15 | 16 | 87 | 131 | 44.8 | 85.3 | 46.4 | 84.5 | 76.3 | 45.6 |

**Supplemental References**

1. Karatzoglou A, Meyer D, Hornik K. Support vector machines in R. Journal of statistical software 2006;15:1-28.

2. Tong S, Koller D. Support vector machine active learning with applications to text classification. Journal of machine learning research 2001;2:45-66.
